# GREEN-DB: a framework for the annotation and prioritization of non-coding regulatory variants from whole-genome sequencing data

**DOI:** 10.1093/nar/gkac130

**Published:** 2022-03-02

**Authors:** Edoardo Giacopuzzi, Niko Popitsch, Jenny C Taylor

**Affiliations:** Wellcome Centre for Human Genetics, University of Oxford, Oxford OX3 7BN, UK; National Institute for Health Research Oxford Biomedical Research Centre, Oxford OX4 2PG, UK; Wellcome Centre for Human Genetics, University of Oxford, Oxford OX3 7BN, UK; Max Perutz Labs, University of Vienna, Dr. Bohr-Gasse 9, 1030 Vienna, Austria; Wellcome Centre for Human Genetics, University of Oxford, Oxford OX3 7BN, UK; National Institute for Health Research Oxford Biomedical Research Centre, Oxford OX4 2PG, UK

## Abstract

Non-coding variants have long been recognized as important contributors to common disease risks, but with the expansion of clinical whole genome sequencing, examples of rare, high-impact non-coding variants are also accumulating. Despite recent advances in the study of regulatory elements and the availability of specialized data collections, the systematic annotation of non-coding variants from genome sequencing remains challenging. Here, we propose a new framework for the prioritization of non-coding regulatory variants that integrates information about regulatory regions with prediction scores and HPO-based prioritization. Firstly, we created a comprehensive collection of annotations for regulatory regions including a database of 2.4 million regulatory elements (GREEN-DB) annotated with controlled gene(s), tissue(s) and associated phenotype(s) where available. Secondly, we calculated a variation constraint metric and showed that constrained regulatory regions associate with disease-associated genes and essential genes from mouse knock-outs. Thirdly, we compared 19 non-coding impact prediction scores providing suggestions for variant prioritization. Finally, we developed a VCF annotation tool (GREEN-VARAN) that can integrate all these elements to annotate variants for their potential regulatory impact. In our evaluation, we show that GREEN-DB can capture previously published disease-associated non-coding variants as well as identify additional candidate disease genes in trio analyses.

## INTRODUCTION

The precise spatiotemporal control of gene expression plays a fundamental role in developmental processes and cellular functions and consequently, is essential in determining human phenotypes (1–3). Gene expression is controlled by the interaction of distal regulatory elements, such as enhancers and silencers, with proximal gene promoters, and is mediated by complex networks of transcription factors (TF) binding to these genomic regions (4–7). Sequence variants within these regulatory regions can alter TF binding and/or enhancer-promoter interactions, resulting in gene expression dysregulation and eventually disease (8–13). The contribution of regulatory regions in human diseases is also supported by a myriad of genome-wide association studies (GWAS), showing that most disease-risk variants lie in non-coding regions (14–16). In recent years, our knowledge about regulatory mechanisms and regulatory elements across the human genome has substantially improved due to a large number of genomic, epigenomic, and transcriptomics studies. Main functional elements in the human genome, such as enhancers, promoters, and TF binding sites, have been extensively mapped by large international collaborations like ENCODE (17,18) and FANTOM5 (19,20). Several dedicated resources have subsequently been developed, integrating and extending these datasets to generate a more detailed picture of regulatory elements (21–26). Meanwhile, the application of novel computational (26–29) and high-throughput screening methods (30–33) has substantially improved our understanding of how regulatory elements control their respective target genes while several *in-silico* methods have been developed to better predict the impact of non-coding regulatory variants (34–40).

The increasing adoption of whole-genome (WGS) over whole-exome (WES) sequencing in disease studies now allows for the comprehensive investigation of human variants (41), including those affecting these regulatory regions. The accurate identification, interpretation and prioritization of such WGS-derived variants requires standardized resources for their annotation in routine bioinformatics pipelines in order to identify likely pathogenic regulatory variants. Whilst there is a large variety of annotation methods and databases available for coding variants (42,43), resources for programmatic annotation of regulatory variants and their respective target gene(s) are still lacking. Ideally, such resources would include a catalogue of regulatory regions and functional elements together with a set of impact prediction scores (40,44). However, the resources currently available in this field are often presented in a format not suitable to this task, and information about controlled gene(s) and tissue(s) of activity is difficult to access programmatically.

To fill this gap, we have created a comprehensive resource for regulatory variant annotation including a collection of ∼2.4M regulatory elements, GREEN-DB (Genomic Regulatory Elements ENcyclopedia Database); additional functional elements (TFBS, DNase peaks, ultra-conserved non-coding elements (UCNE), topologically associating domains (TADs) and super-enhancers) and pre-processed prediction scores. Information on the controlled gene(s), tissue(s), and associated phenotype(s) are provided in GREEN-DB when possible. Here, we present a unified framework that can leverage this new resource to process standard variant call format (VCF) files and generate a comprehensive annotation of non-coding variants. We anticipate that this will aid annotation of regulatory non-coding variants identified from WGS thereby improving variant interpretation and diagnostic yield.

## MATERIALS AND METHODS

### Data collection

To compile an up-to-date, comprehensive collection of regulatory elements in the human genome (GREEN-DB) we collected and aggregated information from 16 different sources, including seven previously published curated databases, six experimental datasets from recently published articles, and predicted regulatory regions from three different algorithms. Five additional datasets were included to integrate region to gene/phenotype relationships. The full list of data sources and references is reported in Supplementary Table S1. We also collected additional data useful in evaluating the regulatory role of genomic regions, including TFBS, DNase peaks, ultraconserved non-coding elements (UCNE), super-enhancer definitions, and enhancer LoF tolerance (Supplementary Table S2) as well as 28 scores developed to predict the regulatory impact of non-coding variants (Supplementary Table S3).

### Data processing

Original tables from the various data sources were processed to generate a normalized representation of potential regulatory regions organized in a SQlite database containing information on their controlled gene(s), method(s) of detection, tissue(s) of activity and associated phenotype(s). When needed, region coordinates were converted from GRCh37 to GRCh38 coordinates using the UCSC LiftOver tool. We performed several processing and quality control steps to remove unreliable regions and to reduce information redundancy by collapsing regions with large overlap. For this clean set of regions we then used information from GTeX eQTLs to infer additional regulated genes, and large GWAS catalogues and HPO annotations to infer phenotypes associated with each region. Finally, we refined the region-to-gene connections to ensure consistency in gene representation and remove connections with poor support. Additional datasets such as TAD domains, TFBS, DNase clusters, super-enhancer, UCNE, and enhancer LoF tolerance were also included in GREEN-DB with information on their overlap with the regulatory regions. A detailed description of the extensive data processing steps that were undertaken, and the SQlite database organization is provided in Supplementary Methods.

### Evaluation of collected regulatory regions

Given that creation of the GREEN-DB regions table involved a complex pre-processing of the original data sources, we first verified if these regions can still capture functional and conservation signals, features which are often reported to provide support for the regulatory role of variants in the original data sources. First, we used Fisher's exact test to evaluate over-representation of functional genome elements in GREEN-DB regions considering ENCODE TFBS, ENCODE DNase hyper-sensitivity clusters, UCNE regions, and a curated set of non-coding disease-associated variants (from (36)). Then, we also investigated the overlap with genomic low-complexity regions (as defined in (45)) and segmental duplications, which are mostly uninformative for variant detection. Finally, we generated a set of control regions by randomly picking from each chromosome (excluding centromeric and telomeric regions) the same number of regions seen in GREEN-DB, with comparable size distribution (Supplementary Figure S1), and compared the degree of conservation and the distribution of prediction scores between these regions and the GREEN-DB regions. Using the Mann–Whitney *U* test, we compared the fraction of bases having a PhyloP100 score above 1, 1.5 and 2 (higher values indicate greater conservation) and compared the median and maximum score values for ncER, FATHMM MKL and ReMM scores obtained for the GREEN-DB and control regions. We also performed an in-depth analysis of the gene-region connections collected in the database, evaluating the distance between a region and its controlled genes, the occurrence of connections within TAD domains and the specificity of gene-region relationships (see Supplementary Methods). We then compared information in GREEN-DB with information present in each of the 16 individual data sources that were collected in our database and for each dataset we computed the fraction of genome covered and the number of genes present in the dataset annotations.

### Identification of regions under variation constraint

We used data from gnomAD v3 to evaluate the possible variation constraint across GREEN-DB regions by evaluating the deviation of observed number of variants from the expectation based on a linear regression model including region length, GC percentage and overlaps with segmental duplications, low-complexity regions and exonic regions. Regions above the 99th percentiles of the resulting constraint value were considered as constrained regions. We characterized these regions and the controlled genes by looking at: (i) possible enrichment of true positive variants in the curated set of disease-causing non-coding variants from (36), Gene Ontology groups, canonical pathways, essential genes, and ClinVar pathogenic genes; (ii) distribution of the highest constraint value for each gene present in GREEN-DB; (iii) maximum constraint value between regions controlling genes in the ClinVar pathogenic or essential genes groups and all other regions in GREEN-DB. More details are described in Supplementary Methods.

### Evaluation of non-coding impact prediction scores

With the aim of providing a framework useful for variant prioritization, we reviewed the potential of 28 non-coding variant impact prediction scores to inform the analysis of WGS data from patients with rare diseases. Among these, we excluded: 10 scores not providing pre-computed values, making them difficult to apply programmatically, 2 scores developed specifically for somatic variants, and 1 score providing only disease-specific predictions for a limited set of phenotypes (see Supplementary Table S3). We used the ROCR package (46) to compare the classification performances of the remaining 19 scores when applied to a set of curated disease-causing non-coding variants from (36) including 725 true positive examples and 7250 negative examples. More details are given in Supplementary Methods.

### Prioritization strategy for regulatory variants using GREEN-VARAN

Our GREEN-VARAN tool ranks variants overlapping GREEN-DB regions from Level 1 to Level 4 by combining GREEN-DB data with population allele frequency, impact prediction scores (ncER, FATHMM MKL, ReMM), and functional elements (TFBS, DNase peaks, UCNE) as detailed in Table 1. These four levels sum up information that supports the regulatory impact of variants located in GREEN-DB regions so that variants with higher levels are supported by multiple types of evidence (i.e. low allele frequency in general population, high prediction scores, overlapping functional elements, constrained regulatory region). After variant prioritization, to further assist in result interpretation, the resulting candidate genes are ranked based on patient HPO profiles using GADO (47). Based on a list of HPO terms, GADO uses a pre-trained prediction matrix to return a ranked list of all human genes annotated with *Z*-scores that summarize the predicted relationship of each gene with the provided HPO profile. Using GADO, we considered genes above the 95th and 99th percentile in the GADO ranking as likely or strongly disease-related, respectively.

**Table 1.** Criteria used in the prioritization strategy for non-coding variants. This is based on GREEN-VARAN variant classification complemented by HPO-based ranking of candidate genes.

Level	Criteria
Level 1	Rare variant (population AF < 1%) overlapping one of GREEN-DB regions
Level 2	Level 1 + overlap with at least one functional element among transcription factors binding sites (TFBS), DNase peaks, ultra-conserved elements (UCNE)
Level 3	Level 2 + prediction score value above the suggested FDR50 threshold for at least one among ncER, FATHMM-MKL, ReMM
Level 4	Level 3 + region constraint value ≥0.7
HPO-based prioritization	Level 4 + linked to a gene in the top 90th percentile of HPO-based ranking according to GADO prediction

### Application to WGS trio analysis

We applied this strategy to analyse 53 WGS trios with recessive mode of inheritance and computed the resulting number of recessive and compound heterozygotes candidate variants when considering only coding variants, only GREEN-DB annotated variants or the combination of both. In each scenario, we computed the number of candidates considering either all genes, genes prioritized based on the HPO profiles using GADO (see prioritization strategy above), clinically relevant genes from ClinVar pathogenic list, and PanelApp disease genes. Details on the WGS cohort and the filtering strategy are given in Supplementary Methods.

### Evaluation using validated disease-causing non-coding variants

To evaluate the performance of the proposed prioritization method, we applied it to an independent set of the 45 rare disease-causing non-coding variants described in (48) (Supplementary Table S4). These variants were selected since they are independent from those used in most of the considered prediction scores. First, we computed the number of variants captured at each of the four prioritization levels. Then, we spiked each validated variant into the full set of WGS variants obtained for the reference sample NA12878 and evaluated the number of possible candidate variants resulting at each level assuming the disease-causing gene was known. Finally, to better assess the ability of the proposed method to reduce the number of possible candidates and prioritize the correct causative variant among all variants identified in a patient genome, we generated an HPO profile for each simulated genome by randomly sampling a maximum of 5 HPO terms associated with the relevant disease and computed the number of candidate variants resulting from GREEN-VARAN annotations using GADO to prioritize disease-related genes. In each case, we also investigated where the known causative variants were ranked compared to all other candidate variants based on the GADO *Z*-score. We repeated this analysis assuming either a recessive or dominant mode of inheritance. To better evaluate how GREEN-DB annotations can help identifying regulatory variants in distant control elements, we also applied our annotations to a set of 18 previously published variants involved in human diseases and located in distant enhancers with a validated effect on gene expression (Supplementary Table S5). For both, the set of 45 rare disease-causing non-coding variants and the set of 18 variants located in distal enhancers, we compared how many variants can be captured by GREEN-DB and by each one of the 16 data sources collected in the database. More details are given in Supplementary Methods.

### Comparison with Genomizer prioritization

We compared our GREEN-VARAN prioritization approach with Genomizer (38), a previous framework for WGS variant analysis applicable to non-coding variants. The Genomizer tool takes WGS variants, a set of HPO terms and a PED file as input and applies an automated annotation and filtering process to return a list of candidate variants annotated with a variant-based score, a gene-based score and a combined score that summarizes both. We applied Genomizer v12.1.0 (using data v21_09, CADD score v1.6 and ReMM score v0.3.1) to the same set of simulated genomes and HPO-profiles described above using the default filtering parameters for whole-genome analysis, which includes a regulatory region filter and the use of CADD and ReMM prediction scores. We then compared how many of the 45 variants in the validated non-coding variant set can be captured and associated with the correct gene by Genomizer and by GREEN-VARAN annotations and how many appear in the top 10 genes ranked by Genomizer and in the level 3 variants selected by GREEN-VARAN. Additionally, we evaluated for each of the 45 simulated genomes, how many candidate variants were retrieved for the known disease gene by both methods, considering GREEN-VARAN level 1 variants and all variants filtered by Genomizer. We also compared the two methods in a scenario where the disease gene is not known, evaluating how many variants were filtered by GREEN-VARAN at each prioritization level compared to all variants filtered by Genomizer and compared how the known causative variant was ranked by each method when HPO-based prioritization was also used. The ranking was based on the combined score for Genomizer and on the GADO *Z*-score for GREEN-VARAN. Finally, to assess the performance of the two methods considering more challenging regulatory variants in distant control elements, we applied the Genomizer pipeline to the set of 18 previously published variants involved in human diseases and located in distant enhancers. We then compared the number of variants filtered by GREEN-VARAN at each prioritization level and by Genomizer and how many of them were associated with the correct gene.

## RESULTS

### The GREEN-DB database

**Figure 1.**
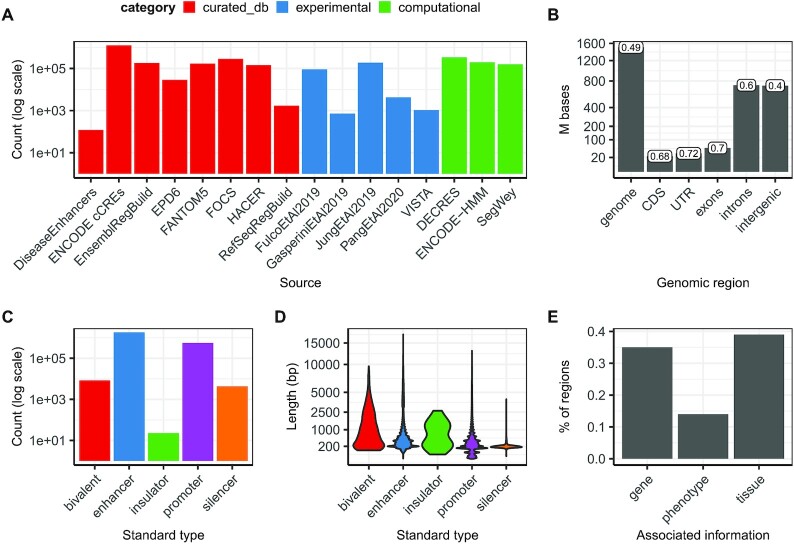
Summary statistics for regions collected in the GREEN-DB. (**A**) GREEN-DB collects human regulatory regions from 16 different sources including curated databases, experimental assays, and computational predictions. (**B**) Number of bases captured by these regions across different genomic locations and covered fraction of each genomic location (label on top of bars). (**C**) GREEN-DB contains bivalent, enhancer, insulator, promoter and silencer regions with sizes mostly between 100 and 1000 bp (**D**). (**E**) Fraction of regions with associated gene, phenotype and tissue information. Phenotype information was derived from GWAS studies (via overlap of significant SNPs with GREEN-DB regions), HPO (via controlled genes) and DiseaseEnhancer dataset.

We have created a comprehensive collection of potential regulatory regions including ∼2.4M regions from 16 data sources covering ∼1.5Gb in the human genome (Figure 1A, B and Supplementary Figure S2). This corresponds to a ∼49% coverage across the genome, an increase of ∼14% compared with the largest data source among the ones collected in the database (Supplementary Figure S3A). A summary of the regions present in GREEN-DB is given in Table 2 with more details in Supplementary Table S6. Overall, these regions cover ∼60% of introns and ∼40% of intergenic space (Figure 1B), but overlap was observed also with UTR and other exonic regions (Supplementary Figure S4 and Supplementary Tables S7, S8). We grouped regulatory regions into five categories: bivalent (regions showing both activation and repression activity), enhancer, insulator, promoter, silencer; with enhancer and promoters representing the majority of regions (Figure 1C) with size distributions shown in Figure 1D. Each region is described by its genomic location, region type, method(s) of detection, data source and closest gene/TSS and ∼35% of regions are annotated with controlled gene(s), ∼40% with tissue(s) of activity and ∼14% have associated phenotype(s) (Figure 1E). These data are organized in an SQLite database allowing for rapid querying based on genomic interval(s) and/or gene(s) of interest (the database structure is described in Supplementary Results and in Supplementary Figure S5). GREEN-DB regions are also provided as extended BED files for integration into existing analysis pipelines.

**Table 2.** Summary of GREEN-DB information, reporting regions counts and number of genomic bases covered

	GRCh37			GRCh38		
GREEN-DB	No. of elements	Mean size (bp)	Bases covered	No. of Elements	Mean size (bp)	Bases covered
Enhancer	1 834 183	1107	1 450 755 698	1 832 830	1107	1 449 153 178
Promoter	566 102	573	234 890 654	565 323	573	234 315 553
Silencer	4306	208	895 868	4302	208	894 792
Bivalent	8413	1333	11 215 000	8409	1333	11 210 309
Insulator	23	846	17 504	23	846	17 504
All regions	2 413 027	981	1 504 116 499	2 410 887	981	1 502 180 018

Given that creation of the GREEN-DB regions table involved a complex pre-processing of the original data sources, we first verified whether our database regions can replicate the overlaps with functional and conservation signals reported in the original data sources. Processed regions in GREEN-DB maintain strong support for a regulatory role as indicated by the enrichment of several functional genomic signals including transcription factor binding sites (TFBS, OR 9.67), DNase hypersensitivity peaks (OR 13.13), GTeX significant eQTLs (OR 2.93), ultra-conserved non-coding elements (UCNE, OR 8.35). At the same time, they are, as expected, depleted for difficult-to-address regions such as segmental duplication (SegDup, OR 0.45) and low-complexity regions (LCR, OR 0.22). Finally, they are also enriched for a curated set of non-coding disease-causing mutations (OR 2.05) (Supplementary Figure S6A and Supplementary Table S9). Compared to random regions with comparable size and distribution across the genome, GREEN-DB regions showed a larger proportion of bases with PhyloP100 score above 1, 1.5 and 2 (*P*-value < 2.2E–16 for all comparisons, Mann–Whitney *U* test) (Supplementary Figure S6B) as well as higher per-region median and maximum values for ncER, FATHMM MKL and ReMM scores (*P*-value < 2.2E–16, Mann–Whitney *U* test) (Supplementary Figure S6C).

### Controlled genes annotation in GREEN-DB

**Figure 2.**
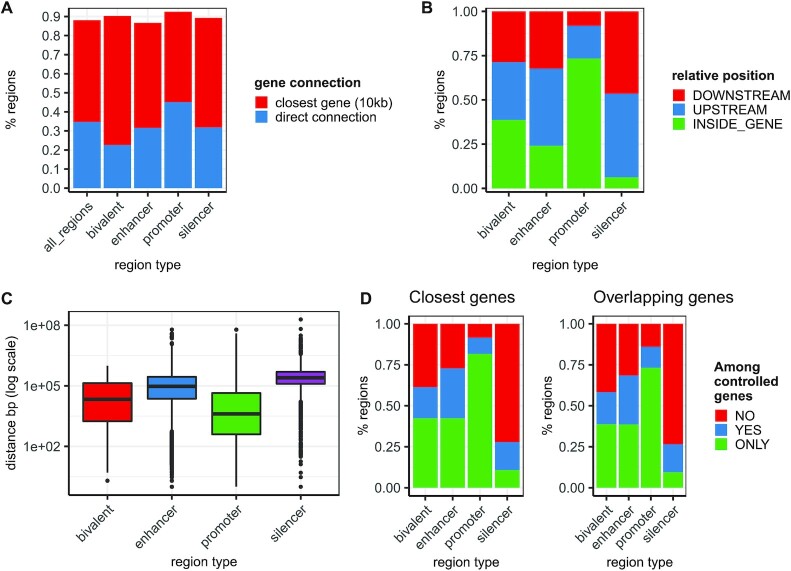
Gene regulatory space. (**A**) Overall 839 807 regions (∼35%) in GREEN-DB are experimentally associated with a controlled gene, and the fraction of regions with a plausible associated gene reaches ∼90% when we include close genes within 10 kb distance. (**B**) Considering only experimental associations, distant control elements are mostly located up- or downstream of a gene, with a smaller proportion observed within genes. The large proportion of gene overlap observed for promoters is mostly explained by their partial overlap with the first gene exon. (**C**) The distance between a region and its controlled gene(s) is larger for enhancers, silencers and bivalent, than for promoters, with most regions located between 10 kb and several Mb away from the controlled gene. (**D**) Interestingly, a large proportion of these regions may not control their closest gene(s) even when they are located within a specific transcript.

For each region in the database we annotated controlled genes based on experimental screens or previously curated collections (directly controlled genes), the closest gene and the closest transcription starting site (TSS). Overall, about 35% of GREEN-DB regions have at least one directly controlled gene, ranging from 22% for bivalent regions up to 45% for promoters (Figure 2A). An additional 36% of promoter regions have a TSS in close proximity (distance ≤ 10 kb), so that a controlled gene can be confidently assigned to 81% of promoters. Considering other types of regions, we observed a close gene or TSS within 10 kb for ∼55% and ∼25% of regions without a directly controlled gene, respectively.

Considering only the 837 879 regions annotated with directly controlled genes, they interact with a total of 48 230 different genes, covering 67% of all genes and 97% of protein-coding genes from ENCODE v33 basic set. Controlled genes also cover 97, 98 and 100% of clinically relevant genes from PanelApp (49), ClinVar (pathogenic genes only) and ACMG actionable genes list, respectively (Supplementary Table S10). This represents a notable increase in gene-specific information compared with the genes annotated in the single data sources collected to create GREEN-DB (Supplementary Figure S3B). Considering directly controlled genes, the associated enhancer and silencer regions can be located either upstream or downstream to the controlled gene at a distance ranging from 10 kb up to a few Mbs (Figure 2B, C). We also observed that the closest gene is among annotated controlled genes only for ∼70% of enhancers and ∼25% of silencers, while this proportion is much higher (∼90%) for promoters, as expected. The closest gene is not the only one controlled in 10–25% of cases for distal control elements, even when the element overlaps a specific gene (Figure 2D). The region-to-gene relationships showed a high degree of specificity, with most regions controlling less than five genes, while most genes are controlled by multiple regions (Supplementary Figure S7). Most regions are also active in a limited number of tissues and the number of controlled genes correlates with the number of tissues in which the region is detected (Supplementary Figure S8). Finally, we observed that genes with a large regulatory space are enriched for pathogenic and essential genes (Supplementary Table S11). Additional details on the genes’ regulatory space are provided in Supplementary Results.

### Regions constrained against sequence variation

We calculated a constraint metric for GREEN-DB regions ranging from 0 to 1, so that regions with higher values have lower than expected numbers of variants. Based on this metric we defined as constrained the 23 102 regions above the 99th percentile of the distribution (mostly enhancers and promoters, Supplementary Figure S9). Comparison with other regions in GREEN-DB showed that constrained regions are more conserved (Supplementary Figure S9C) and enriched for tissue or gene-specific regions (*P* < 2.2E–16, Supplementary Figure S10) and for true disease-causing mutations from the curated set (OR 13.32, 95% CI 8.32–21.55, *P* 1.42E–27).

**Figure 3.**
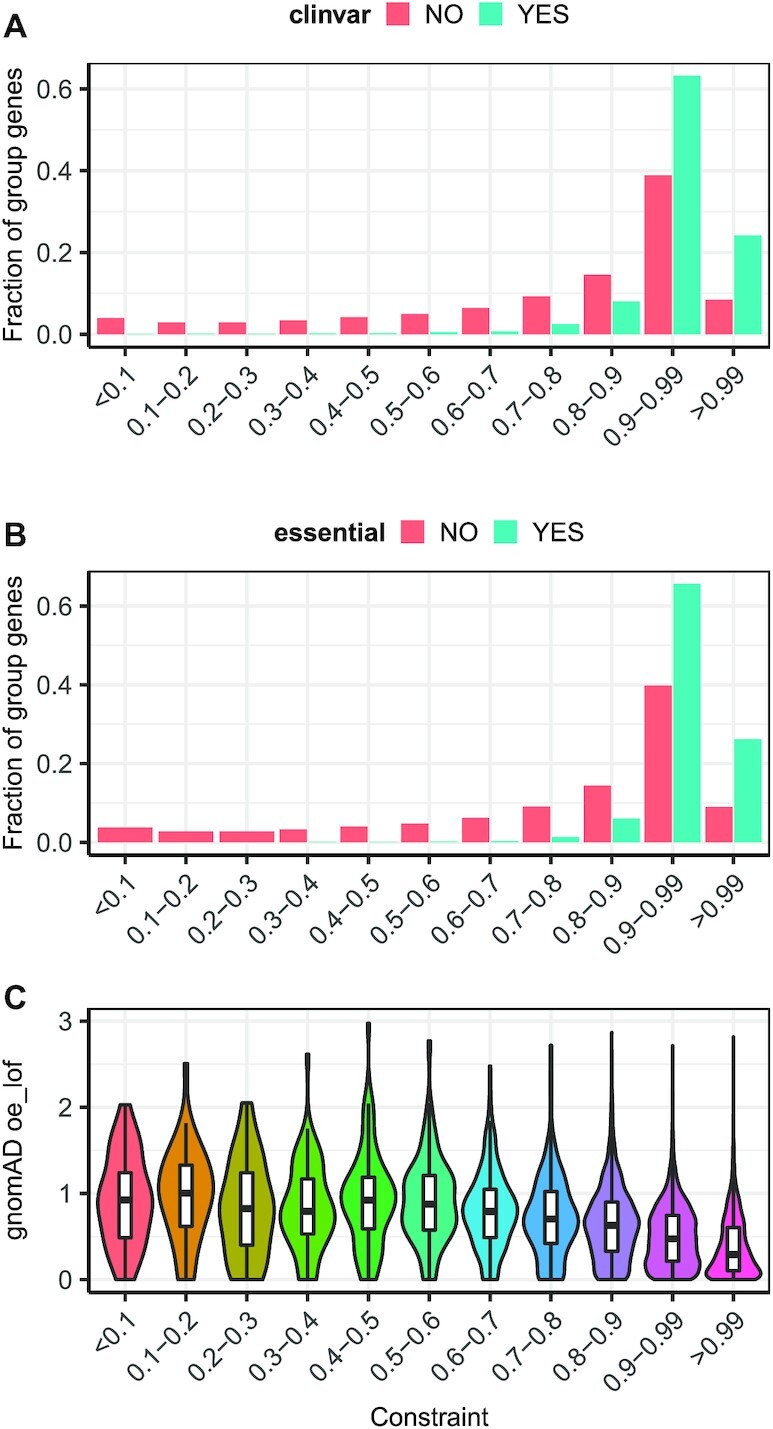
Constraint regions control diseases-associated and essential genes. For various constraint value tranches, we calculated the fraction of ClinVar (**A**) or essential (**B**) genes from mouse knockout screens controlled by at least one region in the corresponding tranche. Both groups show a large fraction of genes controlled by regions with constraint value ≥0.9 and >95% of genes in each group are linked to a region with constraint ≥0.7. Regions with high constraint are also controlling genes with lower oe_lof value in gnomAD (**C**), suggesting they are associated with genes intolerant to variations.

Overall, constrained regions control 4579 genes and these genes are strongly enriched for essential genes and genes bearing pathogenic variants in ClinVar (FDR 4.53E–133 and 6.21E–198, respectively). Complete enrichment results are reported in Supplementary Table S12. When comparing the maximum constraint value of associated GREEN-DB regions, genes in the ClinVar pathogenic and essential groups are controlled by regions with higher constraint value compared to other genes (*P* < 2.2E–16, Matt–Withney *U* test, Supplementary Figure S11A, B). Overall, regions above the 70th percentile control >95% of ClinVar pathogenic genes and essential genes and are associated with genes with lower observed/expected ratio for loss of function variants in GnomAD (oe_lof) (Figure 3). However, the median constraint value considering all associated regions is only slightly higher for pathogenic and essential genes compared to normal genes (Supplementary Figure S11C, D).

### Evaluation of non-coding impact prediction scores

We considered 28 previously published prediction scores that can be applied to evaluate the impact of non-coding variants. Of these, 13 do not provide pre-computed values or were developed for somatic variants only and were thus removed from further analyses, while GWAVA and EIGEN provide 3 and 2 possible values respectively. Using a curated set of disease-causing, non-coding variants from (36), we evaluated the performance of 18 scores in classifying disease-causing variants (Supplementary Figure S12). The FINSURF algorithm obtained the best overall classification result (OPM 0.73, AUC 0.94), but the pre-computed scores only cover 15% of the genome, limiting its application in WGS annotation. We selected ncER, FATHMM-MKL and ReMM as the best scores combination that provided both high genomic coverage (> 90%) and good classification performances (OPM > 0.4 and AUC > 0.8) (Supplementary Table S13). Overall, no single score seemed able to robustly remove false-positive calls while maintaining high sensitivity. Indeed, when TPR is set to 0.9, the FDR is >0.5 for all scores, while controlling the FDR ≤0.5 results in TPR values <0.5 for most scores. To assist the use of these scores in variant analysis, we also computed the score thresholds corresponding to TPR ≥0.9, FDR ≤0.5, and maximum accuracy (detailed metrics are shown in Supplementary Figure S13 and Supplementary Table S14).

### A framework for annotation and prioritization of non-coding variants from WGS

We created a tool (GREEN-VARAN: Genomic Regulatory Elements ENcyclopedia VARiant ANnotation) to annotate VCF files with information from GREEN-DB. The tool is written in the Nim programming language using hts-nim (50) and processes standard VCF files by adding annotation on overlapping regulatory region(s) type(s), IDs and constraint values, controlled gene(s) and closest gene(s) with their distance. The tool can also update existing gene consequence annotations from snpEff or bcftools and a tag can be added to highlight variants linked to gene(s) of interest. When allele frequency, non-coding prediction scores and functional element annotations are present, GREEN-VARAN also classifies variants according to the 4 levels described in Table 1. However, the prioritization strategy is fully configurable to be able to take into account additional custom annotations present in the input VCF file. Additional pre-processed datasets useful for annotation and a Nextflow (51) workflow are distributed together with GREEN-DB and can be used to generate a fully annotated VCF for small non-coding variants. The tool is also capable of annotating large variants (CNVs, structural variants), but in this case only the information on the affected regulatory regions and controlled genes are provided for each variant, while variant classification is not provided given that a single structural variant is likely to overlap multiple regulatory regions. Given an annotated VCF, a list of variants or a list of GREEN-DB IDs, GREEN-VARAN can also be used to query GREEN-DB and retrieve detailed annotations including tissue(s) of activity, data source(s) and associated phenotype(s). More details on the tool, available datasets and the annotations added to VCF are given in Supplementary Results.

### GREEN-VARAN annotation captures validated non-coding, disease-associated variants

**Figure 4.**
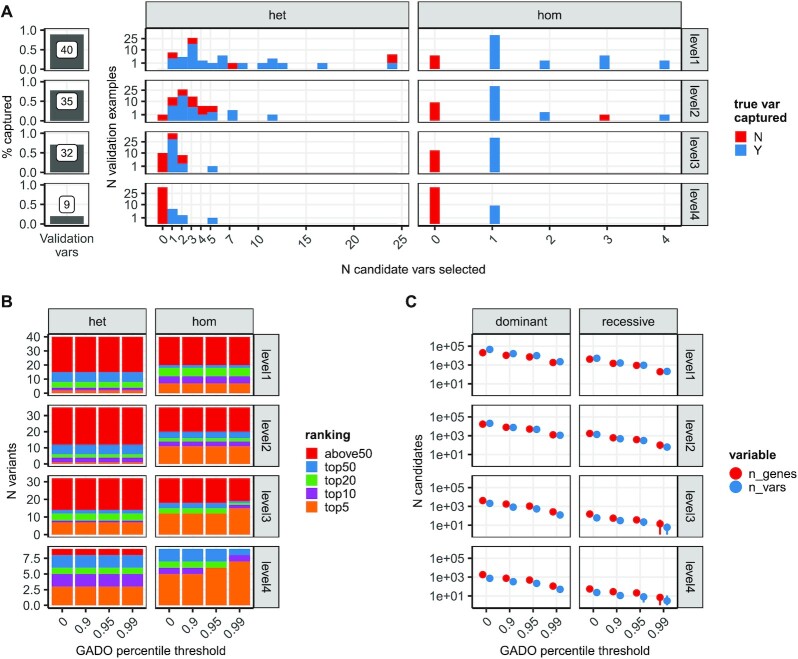
Application of GREEN-VARAN to validated variants. We inserted a set of 45 validated variants into variants from the reference sample NA12878 and tested how GREEN-VARAN annotations are able to capture them and how many candidate variants are selected when considering only the disease-causing gene (**A**). We then evaluated how the known causative variant is ranked compared to all other candidate variants selected by our prioritization method in combination with HPO based gene ranking when the disease gene is not known (**B**). Finally, we computed how many candidate variants/genes are retrieved in each scenario (**C**).

We used an independent set of 45 rare curated variants from (48) (Supplementary Table S4) to evaluate how GREEN-VARAN annotations can help capture disease-causing variants in non-coding regions. When applying the proposed prioritization system, we observed 40 (89%), 35 (78%), 32 (71%) and 9 (20%) validated variants captured at Level 1, 2, 3 and 4, respectively. When we added these known variants into WGS variants from the reference NA12878 sample and looked at variants associated with the relevant gene only, GREEN-VARAN prioritization was able to reduce the number of candidate variants to <25 and <5 for heterozygous and homozygous variants, respectively. The possible candidates were reduced to <5 and just one when considering level three variants, even if 29% of causative variants were lost (Figure 4A). To simulate a more realistic scenario where the disease variant has to be identified without a knowing the disease gene involved, we generated an HPO profile for each simulated genome by randomly sampling a maximum of five HPO terms associated with the relevant disease (Supplementary Figure S14) and integrated GREEN-VARAN annotations with HPO-based gene prioritization by GADO. Using mild filtering (Level 1 variants and 0.9 GADO threshold) this approach generated 13k–23k and 1k–2k candidate variants for dominant and recessive inheritance respectively, while a more stringent filtering (Level 3 variants and 0.95 GADO threshold) reduced the number of candidates to 400–800 and 8–32, while still capturing 71% of the validated examples (Figure 4C, Supplementary Table S15). The correct variant was ranked among the top 10 variants in 11/32 cases (34%) when considering Level 3 variants and 0.95 GADO threshold and 17/32 cases (53%) when considering only genes strongly associated with the disease (0.99 GADO threshold) (Figure 4B).

Most of the above validated variants in non-coding regions are actually located in UTR or intronic regions of the controlled gene; thus we extended our analysis to a set of 18 previously published variants located in distant enhancers with demonstrated regulatory effects on a disease gene. GREEN-DB annotations were able to capture all tested variants, linking them to the expected gene. When applying the proposed prioritization strategy we found 18, 13 and 2 variants ranked at Levels 2, 3 and 4, respectively. Details of each variant are reported in Supplementary Table S5.

When compared with the individual data sources integrated in the database, GREEN-DB annotations captured a larger proportion of variants from our non-coding evaluation datasets, both the set of 45 rare disease-causing non-coding variants as well as the 18 variants located in distal enhancers (Supplementary Figure S15).

### Comparison with Genomizer prioritization

Genomizer and GREEN-VARAN performed similarly when applied to a set of 45 rare disease-causing non-coding variants. When considering all variants identified, both methods were able to capture 40 validated variants and associate them with the correct gene, while 32 and 33 variants were captured when considering GREEN-VARAN level 3 variants and variants at the top of the Genomizer ranking, respectively (Supplementary Figure S16A). When applied to the simulated genomes for the prioritization of WGS variants related to a single specific disease gene, both methods retrieved a similar number of candidates and both resulted in <20 and <5 candidate variants when considering homozygous and heterozygous candidates, respectively (Supplementary Figure S16B). When we simulated a scenario where the disease gene is not known, however, GREEN-VARAN was able to better refine the number of candidate variants, returning <5000 and <1000 variants at level 3 for the dominant and recessive model, respectively, while Genomizer returned >10 000 variants in several cases (Supplementary Figure S16C). When integrated with the HPO-based prioritization step, the Genomizer achieved a better ranking for the 45 causative variants and was able to rank the correct variant in the top 10 in 21 and 33 cases for the dominant and recessive model, respectively, while GREEN-VARAN ranked them in the top 10 in only 8 and 17 cases, respectively (Supplementary Figure S16D). However, when both were applied to a set of 18 previously published variants located in distant enhancers, GREEN-VARAN was able to associate all of them to the correct gene while Genomizer completely failed to capture these associations (Supplementary Figure S17 and Supplementary Table S5).

### GREEN-VARAN annotation reveals new candidate genes in WGS trio analysis

**Figure 5.**
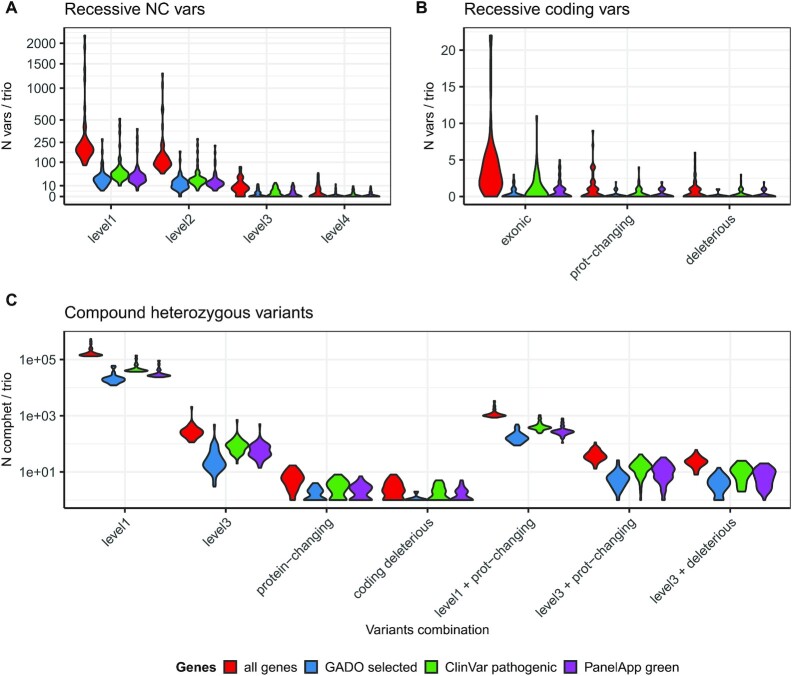
Impact of non-coding annotations on WGS variant prioritization. The violin plots represent the number of recessive candidate variants found in 53 WGS trios for non-coding variants prioritized by GREEN-VARAN (**A**) or considering only coding variants (**B**). We then considered compound heterozygotes combinations (**C**) reporting the number of candidates when considering only GREEN-VARAN prioritized variants (Level 1 and Level 3), only coding variants or combinations involving one coding and one non-coding variants. In all plots the counts are reported for all genes, for genes ranked above the 90th percentile in GADO distribution (GADO selected), for ClinVar pathogenic genes and for PanelApp disease genes.

To evaluate the impact of adding non-coding annotations in a more realistic scenario, we applied our annotation framework to the analysis of small variants from 53 non-consanguineous WGS pedigrees. The main aim was to evaluate the number and relevance of new candidate variants and genes, considering that the interpretation of VUS and novel genes is time consuming and expensive and thus it is important that new tools do not add too many false-positives. Considering variants identified in each individual, we found a median of ∼75.7k rare variants (population AF < 0.01), including ∼38k in GREEN-DB regions (Level 1), ∼17k also overlapping functional signals (Level 2) and ∼1.5k further prioritized based on prediction scores (Level 3) (Supplementary Figure S18). When considering rare recessive variants that segregate with the phenotype in each pedigree, adding GREEN-DB annotations increased the number of candidate variants from 0 to 22 (exonic variants only) to 83 to 2209 (GREEN-VARAN Level 1). Looking at variants with stronger support for regulatory impact, the number of candidates is reduced to 45–1291 at Level 2, 0–75 at Level 3 and 0–47 at Level 4 (Figure 5A, B). When considering compound heterozygotes involving a protein-changing variant we observed 853–3362 combinations with a Level 1 variant and 13–112 with a Level 3 variant (Figure 5C). The new candidates identified also included genes likely to be relevant to the disease phenotype based on HPO profiles. Indeed, when restricting to genes with high GADO score the number of recessive candidates is increased from 0 - 2 (exonic variants only) to 0–9 (Level 3 GREEN-DB variants), and we identified 0–10 new compound heterozygotes involving a protein-changing and a Level 3 variant. A similar trend was observed also when considering only clinically relevant genes from PanelApp or Clinvar. Detailed counts for candidates identified in the trio analysis are reported in Supplementary Table S16.

## DISCUSSION

Variants in non-coding regions of the genome have clearly been implicated in disease risk from both GWAS studies (14–16) and WGS rare disease studies (8,10–12) and the large fraction of missed diagnoses in WES studies (52–54) indicate that the non-coding genome is likely to harbour many variants of clinical diagnostic significance and may account for low diagnostic yield in clinical WGS rare disease studies. As WGS becomes increasingly adopted in research and clinical settings, it becomes critical to have efficient computational tools for the annotation and interpretation of variants in the non-coding genome. Whilst information about types and locations of regulatory regions has previously been described in the literature (17,19–25), the systematic interrogation of these in whole-genome sequencing data remains challenging and limited by the lack of resources to readily access these programmatically (55–57). To fill this gap, we have developed a framework for the automated annotation of WGS variants compiling an extensive catalogue of regulatory regions and developing accompanying tools and resources that can be integrated into routine bioinformatics pipelines to annotate non-coding variants and improve their interpretation and prioritization in rare disease WGS datasets.

We have collected data from published, experimental, and computational sources to create a database providing a standardized representation for ∼2.4 million regulatory elements in the human genome (GREEN-DB) which represents a significant advancement compared to previously published resources. To support the interpretation of the impact of genetic variants, each regulatory region is annotated with a rich set of information including controlled gene(s), closest gene(s), tissue(s) of activity, potentially associated phenotype(s) from GWAS studies and Human Phenotype Ontology, and a constraint metric representing the tolerance to genetic variation. To interpret the biological role of a regulatory region, it is essential to know the genes it controls and in which tissues it is active. GREEN-DB contains experimentally validated region-gene links and tissue information for ∼35% and ∼40% of the regions, respectively. Overall, the database provides regulatory information for 48,246 genes, including most of the clinically relevant genes from PanelApp, ClinVar and ACMG, supporting its usefulness in human disease research.

Our analysis confirms the complexity of the relationship between regulatory regions and controlled genes that can not easily be explained by spatial proximity in the (linear) genome as previously demonstrated, e.g., by high-throughput studies of chromatin interactions (32,58–60). Indeed, for silencer and enhancer elements, the controlled gene was the closest gene in only 10% and 40% of cases respectively, whilst regulatory regions within a gene exert regulatory control on that specific gene in <60% of the cases. Even if we cannot exclude that these observations may be influenced by incomplete annotation of controlled genes, this has considerable implications for the interpretation of non-coding variants where the search for disease-associated genes often starts with the closest genes (61,62). The closest gene and TSS are also annotated for each region so that this information can be used to assign a controlled gene when experimental data is not available. This approach is especially useful for promoters where the close proximity is a strong support for the region-gene interaction, but can also be useful for other kinds of regions. Indeed, despite the fact that we observed that a region/gene link based on proximity would be less certain for these regions, a recent study using UK biobank data showed that genes that are nearest to a trait-associated SNV are more likely to be causative (63). Overall, we observed a high degree of specificity in the region-gene relationship, with a large fraction of regions controlling less than five genes, while most genes are controlled by multiple regulatory regions suggesting that multiple, spatially distant, genomic regions may have similar phenotypic impacts. A correlation emerged between the number of controlled genes and the number of active tissues for each region, confirming the tissue-specific nature of gene regulation and supporting the idea that alterations in a regulatory region can have different impacts in different tissues (12). This makes the comprehensive annotation of all regions that influence or regulate the normal activity of a gene so important for understanding the consequences of genomic variants on the respective gene function.

We also integrated information from GWAS studies and HPO databases to provide a possible associated phenotype for ∼15% of the regions. This resource will be useful for the interpretation of new variants found in the regulatory regions, providing hypotheses on their potential biological impact. The fact that only a limited number of regions has an associated phenotype, despite a large number of GWAS hits available (64–66), can partially be explained by a reduced phenotypic effect of regulatory regions alterations due to complex regulatory network and tissue-specific effects. On the other hand, this also underlines how the impact of rare disrupting variants in the non-coding space is largely unexplored and how resources like GREEN-DB can inform our understanding of human diseases.

We calculated a constraint metric that reflects the tolerance of each region to sequence variations. The maximum constraint value for regions controlling essential genes and genes involved in human diseases is significantly higher compared to other genes and constrained regions (constraint value ≥ 0.99) are associated with genes under variation constraint (low observed/expected ratio for loss of function variants in gnomAD) and strongly enriched for essential genes and genes involved in human diseases. Thus, our constraint metric can be used as a stringent filter to prioritize regions likely to be relevant in controlling disease genes, even if the redundancy of the regulatory network implies that also more variable regions may be involved as suggested by the analysis of median constraint across all regions associated with ClinVar pathogenic genes.

To further assist the interpretation of variants located in regulatory regions, we collected pre-computed values from 19 different impact prediction algorithms and compared their ability to classify a curated set of established disease-causing non-coding variants. Overall, we must take into account that such comparisons are limited by (i) the nature of the known variants collected so far, which are mostly variants within the affected gene and poorly capture distant regulatory elements (57); and (ii) the potential overlap of the test variants with the training sets used by each algorithm, which are often unknown. Based on classification performances and genome coverage, ncER (67), FATHMM (68,69) and ReMM (38) algorithms emerged as the best performing scores, probably reflecting their specific training on disease-associated variants.

We developed a tool (GREEN-VARAN) that integrates information from GREEN-DB, non-coding impact prediction scores, functional elements and population AF annotations, into a 4 level prioritization system to rank the regulatory potential of non-coding variants in a VCF file. The proposed approach can effectively capture variants involved in human diseases, as shown by our ability to recapitulate known disease-associated variants from the literature.

When applied to a set of 45 validated non-coding variants, our approach associated 40 of them with the correct gene and classified 32 as likely impacting gene expression (prioritization level 3), while considerably reducing the number of candidate variants to be evaluated for the causative gene (in 25 cases the causative variant was the only one selected). Since the validated variants we considered were mostly located within the controlled gene, we further tested GREEN-VARAN prioritization on 18 variants in distant enhancers from (70–72) and confirmed its ability to identify the proper controlled gene and prioritize relevant variants (13 out of 18 were classified as Level 3 and likely to be impacting gene expression). While the a priori knowledge of a single candidate gene is unlikely in a rare disease scenario, the rigorous application of a distinctive clinical profile accompanied by clinical tests for known disease genes often enables the identification of a limited number of strong candidate genes. Our results show that GREEN-VARAN is able to restrict associated non-coding candidate variants in such a scenario to reasonably small numbers that can manually be followed-up by clinicians and researchers. When the disease gene is unknown, the integration of GREEN-VARAN prioritization with HPO-based gene ranking, greatly reduces the number of candidates to evaluate in a WGS singleton even if the number of candidates remains challenging (up to 1500 genes) in case of the dominant inheritance model. In the perspective of an increased adoption of WGS by many health systems, this underlines the importance of sequencing also the parents’ genomes so that segregation analysis can be used to reduce the number of candidate variants. In particular, the analysis of a family trio will allow compound heterozygotes to be identified, where a first hit in the coding part of a strong candidate gene is complemented by a non-coding variant affecting the same gene. In this perspective, our approach showed the ability to pinpoint interesting new compound heterozygote combinations in WGS trio analyses.

Application of our new annotation system to a dataset of 53 WGS trios highlighted its potential impact for rare-disease variant analysis. When considering rare recessive variants that segregate with the phenotype in each pedigree, the number of candidate variants is greatly increased by the addition of GREEN-VARAN annotations. However, when considering only variants with stronger support for regulatory impact, the number of candidates is reduced to an amount manageable for downstream analysis (0–75 at Level 3 and 0–47 at Level 4, Figure 4A, B). Whilst this number of candidates is still too many for a diagnostic lab to consider, this is certainly in the realms of the possible for research-based inspection, especially in otherwise difficult-to-solve cases. The new variants prioritized by GREEN-VARAN are of particular interest in compound heterozygote candidates, especially when non-coding variants create new combinations with a prioritized coding variant, an approach that already resulted in increased diagnosis in a recent large clinical WGS study (73). Interestingly, we observed 13–112 combinations with a Level 3 variant and the new candidates identified included genes likely relevant for the disease phenotype based on HPO profiles. A similar trend was observed when considering clinically relevant genes from PanelApp or Clinvar suggesting that inclusion of our non-coding annotation can reveal previously ignored candidate genes likely to have an impact on patient phenotype.

Besides small variants, GREEN-VARAN is also able to annotate regulatory variants affected by large (structural) variants even if the interpretation of the regulatory impact for this kind of variants can be challenging. Indeed, the function of a regulatory element is more likely to be disrupted when hit by a structural variation compared with a single nucleotide variant, but structural variants would often overlap many different elements possibly linked to different genes making the interpretation of the actual biological effect challenging. However, being able to annotate regulatory regions affected by structural variants allows the identification of interesting compound heterozygous combinations, as in the case of a disruptive coding variant complemented by a non-coding SV deleting a regulatory element for the same gene.

Very few solutions exist to prioritize non-coding variants that as well as associate them with putatively affected genes. Compared with Genomiser, a popular automated annotation framework that can run on non-coding variants, our approach showed similar performances in classifying validated non-coding variants close or within a gene. Genomizer achieved a better ranking for the causative variants when using the combined score that also takes into account the HPO-based prioritization of candidate genes, while GREEN-VARAN was able to better refine the number of candidate variants when only variant-supporting evidence was taken into account. Thus, the better Genomizer performance is likely motivated by a better integration of HPO information for known disease genes which has a particular impact given the nature of the tested variants that are associated with well-established disease genes. Moreover, all the variants in the validated set are located within or in close proximity to the causative gene, making it simpler to associate them with the correct gene using a proximity approach. Indeed, when comparing a set of 18 distant enhancer variants, GREEN-VARAN demonstrated a superior ability to prioritize and correctly retrieve the controlled gene, compared with Genomizer, which failed to correctly annotate these variants.

Overall, the proposed framework is not intended for exact variants ranking, but more to provide a summary of evidence for the potential regulatory role of each variant so that they can be more easily interpreted in the context of disease biology. Our approach presents a set of unique features compared to previous methods developed to address non-coding variants or regulatory regions (Table 3). While it provides a summarized view of the regulatory impact support for each variant, it also gives access to a rich set of information useful to enhance variant interpretation and it is able to integrate additional custom annotations that may be available from specific experimental assays like active regions from ChIP-seq or ATAC-seq. Moreover, by helping identify potential regulatory regions of interest for candidate genes our annotations can also be useful to inform the design of further functional experiments.

**Table 3.** Feature comparison between GREEN-VARAN annotations and previous methods

Method	Controlled and closest gene	Tissue of activity	Prediction of variant impact	Easy annotation of VCF files	Integrate multiple supporting evidences	Can use additional custom annotations
GREEN-DB + GREEN-VARAN	X	X	X	X	X	X
Previous non-coding variant ranking systems (e.g. Genomizer, Phen-gen)	Partial (no direct connections for distal control elements)		X	X	X	
Variant impact prediction scores (e.g. CADD, ReMM, LinSight, etc.)			X	X		
Specific regulatory variant prediction scores (e.g. ExPECTO, FINSURF, ncER)	X	X	X	X (but most limited in genome coverage)		
Computational predictions of regulatory elements	Partial (only report closest gene)	X			X	
Previous regulatory region databases	X	X			X	

In summary therefore, we have developed a comprehensive dataset of regulatory regions (GREEN-DB) and integrated it with functional elements and prediction scores into a new framework for the annotation and prioritization of regulatory variants in WGS analysis. We provide a complete annotation workflow implemented in Nextflow that uses our GREEN-VARAN tool to prioritize regulatory variants in VCF files from whole genome sequencing data. The resources presented here therefore represent a significant advance for researchers and clinicians engaged in analysing patient genomes and will be useful for rare disease research as well as for the interpretation of common disease variants from GWAS.

## DATA AVAILABILITY

GREEN-DB BED files and SQlite database are available from https://zenodo.org/record/3981033. GREEN-VARAN tool, the annotation workflow and additional data are available from https://github.com/edg1983/GREEN-VARAN.

## Supplementary Material

gkac130_Supplemental_Files
